# Targeting KRAS in Pancreatic Cancer

**DOI:** 10.3390/jpm12111870

**Published:** 2022-11-08

**Authors:** Darren Cowzer, Mohammed Zameer, Michael Conroy, Walter Kolch, Austin G. Duffy

**Affiliations:** 1 Department of Medical Oncology, Mater Misericordiae University Hospital, D07 R2WY Dublin, Ireland; 2 Systems Biology Ireland, School of Medicine, University College Dublin, Belfield, D04 E1W1 Dublin, Ireland; 3 Conway Institute of Biomolecular & Biomedical Research, University College Dublin, Belfield, D04 E1W1 Dublin, Ireland

**Keywords:** KRAS, pancreas cancer, targeted therapy

## Abstract

Pancreatic cancer is mainly driven by mutations in the KRAS oncogene. While this cancer has shown remarkable therapy resistance, new approaches to inhibit mutated KRAS, KRAS activators and effectors show promise in breaking this therapeutic deadlock. Here, we review these innovations in therapies that target RAS signaling in pancreatic cancer from a clinical point of view. A number of promising approaches are currently in clinical trials or in clinical development. We focus on small-molecule drugs but also discuss immunotherapies and tumor vaccines.

## 1. Introduction

In recent decades, there have been great advances in the treatment of many cancers, whether with immune-based approaches or so-called targeted therapies. One of the main exceptions to this positive trend, however, has been pancreatic cancer (PC), where the main therapeutic advance of the past fifteen years was an intense polychemotherapy combination [1]. Drug development has been difficult and disappointing, and mortality rates for this disease remain very high, even when surgical resection is achievable. RAS oncogenes are the most predominant oncogenes in cancer, causing an estimated 1.5 million deaths globally each year, with varying relative importance within each cancer subtype [2]. Pancreatic ductal adenocarcinoma (PDAC) is driven by oncogenic *KRAS* mutations in at least 80% of cases [3,4]. Along with sustained uncontrollable growth signaling, there is increasing evidence that this alteration mediates autocrine effects and influences crosstalk between immune effector cells within the tumor microenvironment [5]. Oncogenic *RAS* alterations have been shown to result in the suppression of immunity through the modification of the tumor microenvironment with increased immunosuppressive cells, reduced T cell function, the upregulation of immune checkpoints, the downregulation of MHC-I on antigen-presenting cells and altered cytokine production [6,7]. Since the discovery of oncogenes and the birth of the so-called targeted therapy era, RAS has been considered undruggable [8]. The early-phase drug trials in pancreatic cancer have therefore largely focused on the indirect inhibition of RAS, in particular targeting downstream signaling such as the RAF→MEK→ERK pathway, or on the physical tumor microenvironment, including the desmoplastic reaction that is so characteristic of PDAC. These strategies have largely failed, providing no clinically meaningful benefit to patients with PDAC [9,10]. Attempts to understand the escape mechanisms of the RAS effector network through experimental approaches have been frustrated by the dynamic nature of RAS signaling and its multi-branched network of effectors.

## 2. Genomics of PDAC

**Figure 1:**
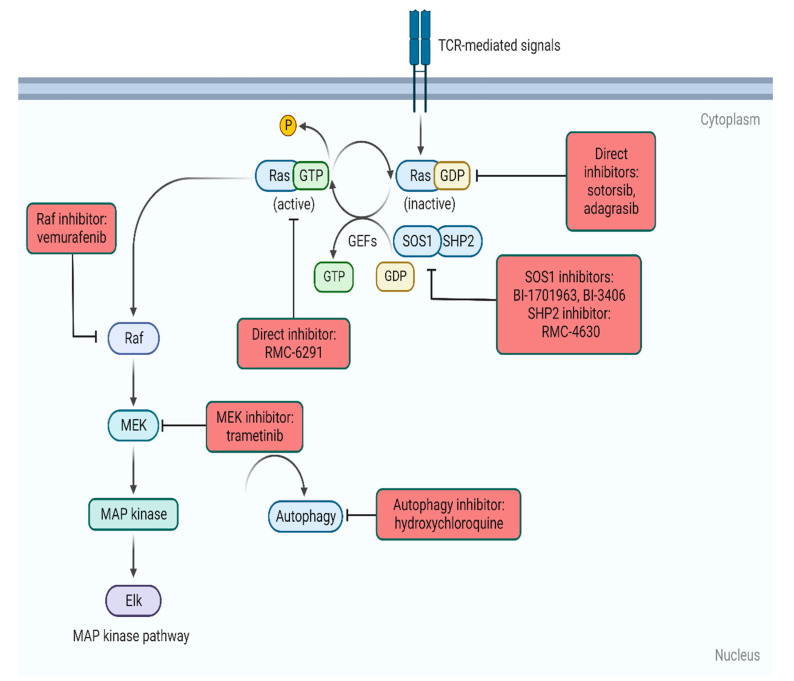
Intracellular targeting of RAS. Created with Biorender.com (accessed on 30 October 2022).

PDAC is a disease not only driven by *KRAS*, but also three other dominant oncogenes—*TP53*, *CDKN2A* and *SMAD4*—which contribute to its biology and treatment resistance [11]. The RAS protein disseminates signals through a network of effector pathways [12]. It receives information from numerous upstream sources—including receptor tyrosine kinases, but also other receptor complexes (including immune cell receptors)—all funneling signals through RAS to drive proliferation via downstream effector pathways, in particular the RAF→MEK→ERK and PI-3 kinase→AKT pathways [12]. The *KRAS* gene encodes a small GTPase which, in the context of cancer, is typically mutated in one of three hotspots, i.e., codons 12 (most commonly), 13 and 61 [13]. These mutations activate the KRAS protein, inducing transforming effects that are enhanced by other cooperative mutations (e.g., loss-of-function TP53 mutations) to spur growth and malignant transformation [14]. Cell populations with *KRAS* gene mutations are not homogenous but vary according to the specific allele or type of mutation, and the allelic distribution varies according to histologic subtype [3]. For example, the most prevalent RAS allele in lung cancer (G12C) reflects the carcinogenic mixture in tobacco smoke that causes that specific change [4]. As we shall see, awareness of the different alleles is of crucial importance for drug development. In pancreatic cancer, the three most common mutations are G12D (33–52%), G12V (23–36%) and G12R (11–20%) [15,16,17] (Figure 1). As we will discuss, the early success with direct RAS inhibitors specifically addressed G12C mutations, which account for only approximately 1% of pancreatic cancer mutations.

## 3. Methods of KRAS Inhibition

### 3.1. KRAS G12C-Specific

The very high affinity of KRAS for GTP has long been considered the main barrier to the development of KRAS inhibitors. This affinity for GTP is much higher than, for example, the affinity of a kinase for ATP, prohibiting the design of competitive nucleotide binding inhibitors that was so successful for kinases. In addition, until recently, no other apparent drug-binding pockets could be found in the structure of RAS proteins [18]. The RAS GTPase cycles between an active (GTP-bound) and an inactive (GDP-bound) state like a switch. The G12C allele was thought to be the most likely druggable allele, as the cysteine residue is amenable to covalent modification [19].

A series of inhibitors were developed which were able to achieve this [20,21], culminating in the first reports of clinical activity with sotorasib and adagrasib. These irreversible *KRAS^G12C^* inhibitors occupy what is termed the switch II pocket and target the inactive, GDP-bound RAS state [22,23,24,25]. The earliest clinical data with direct RAS inhibition were with the covalent inhibitors of the G12C mutation, which accounts for approximately 13% of all KRAS-driven cancers. These drugs target the GDP-bound ‘off’ form of KRAS.

The initial clinical study (CodeBreaK 100) was a phase I/II study evaluating sotorasib (AMG510), and, at initial publication, reported one partial response out of N = 11 patients with PDAC [26]. These data were subsequently updated at ASCO 2022 and reported a response rate of 21% in N = 38 patients with PDAC [27]. These data—which represented an important proof of principle—led to the FDA approval of sotorasib (for KRAS^G12C^) for patients with advanced non-small-cell lung cancer (NSCLC) who harbor a *KRAS^G12C^* mutation and have progressed on at least one prior therapy. Data were recently presented at ESMO 2022 for the phase III study evaluating sotorasib versus docetaxel in patients with *KRAS*^G12C^-mutated NSCLC who had progressed after prior platinum-based chemotherapy and a checkpoint inhibitor. The primary PFS endpoint of the study was met (hazard ratio 0.66; 95% confidence interval [CI] 0.51–0.86; *p* = 0.002), but overall survival was not different. These were subsequently followed by data for another G12C-specific inhibitor (adagrasib), with N = 5 patients experiencing an objective response out of 10 *KRAS^G12C^* PDAC patients treated [28]. Other KRAS^G12C^-specific agents such as LY3537982 have been shown in preclinical models to inhibit GTP loading, with a lower IC50 when compared to other agents [29]. This compound has since entered early-phase clinical testing (NCT04956640).

Overall, the direct inhibition of KRAS appears to be reasonably well tolerated. The most evidence for evaluating toxicity comes from the randomized phase III CodeBreaK 200 study [30]. In this study, where 169 patients received sotorasib grade ≥3, treatment-related adverse events occurred in 33.1%. The most common adverse event was diarrhea (any grade 33.7%), followed by nausea (any grade 14.2%) and increased liver enzymes (any grade 10.1%). Treatment discontinuation due to toxicity occurred in 9.5% of patients.

A comprehensive genetic analysis of patients who have ultimately progressed on KRAS^G12C^ inhibitors has identified a number of different alterations, including other mutations in RAS and RAF pathway proteins, along with amplifications of KRAS^G12C^ alleles and oncogenic fusions that allow the re-activation of the RAF→MEK→ERK pathway [31]. Acquired *KRAS* alterations leading to resistance included G12D/R/V/W, G13D, Q61H, R68S, H95D/Q/R, and Y96C. The mechanisms of resistance acquired that bypass the KRAS^G12C^ target included *MET* amplification; activating mutations in *NRAS*, *BRAF*, *MAP2K1* and *RET*; and oncogenic fusions involving *ALK*, *RET*, *BRAF*, *RAF1* and *FGFR3* [31]. Knowledge of these resistance mechanisms has led to combinatorial strategies in an effort to attain durable responses. Such combinations include the co-inhibition of the EGFR pathways with afatinib and cetuximab, as well as immune checkpoint inhibition with the anti-PD-1 therapy pembrolizumab. 

### 3.2. Other Allele-Specific ‘off’ Inhibitors

For PDAC, in addition to understanding and circumventing emerging resistance in those rare patients who have a G12C mutation, the clear need is for drugs which target the more common mutations. Developing allele-specific inhibitors for the more common mutation types, e.g., *KRAS^G12D^*, is very challenging. The inhibitory activity of the G12C inhibitors relies on the formation of a stable covalent bond between the drug and the reactive mutant cysteine residue. In other alleles, such as the common *KRAS^G12D^*, a reactive residue adjacent to the switch II pocket is lacking, and thus novel approaches toward the identification of selective inhibitors are required. Other allele-specific ‘off’ inhibitors have entered clinical development. For example, Wang et al. recently reported the discovery and characterization of an allele-specific G12D non-covalent inhibitor (MRTX1133)—discovered through an extensive structure-based activity-improvement search—and showed it to be efficacious in *KRAS^G12D^*-mutant implanted cell lines and xenografts [32]. Pursuing the allele-selective approach in general is attractive, as it is likely to be compatible with a wider therapeutic index with more achievable and sustained target inhibition in the clinic, which may be crucial if they are to be used in combination with other treatments, such as chemotherapy or other modalities. A critical advantage of the mutant-specific targeting approach is that there is no inhibition of wild-type RAS-mediated signaling and, therefore, no effect on normal cells (including immune cells) and less toxicity. Whilst toxicities may occur, they are unlikely to be on-target toxicities, because the mutated alleles are not likely to be anywhere in the body apart from tumor cells.

### 3.3. RAS ‘on’ Inhibitors

The ‘off’ inhibitors only work when the mutant RAS protein is still able to cycle through the GDP-bound inactive state and are ineffective when RAS is GTP-bound. Additionally, the effective targeting of G12C has been more fruitful, as it cycles relatively quickly between GDP and GTP binding compared to the other alleles [13]. As with all targeted therapies, emerging resistance is a huge concern. It was noteworthy that, published in the same issue as the initial study demonstrating the clinical activity of sotorasib, there was a paper highlighting multiple mechanisms of resistance to adagrasib [31,33]. The ‘off’ inhibitors may be more susceptible to emerging resistance because of the upstream pressure that may promote new, second-site *RAS* mutations, which can prevent drug binding or the activation/conversion of the wild-type allele to mutant form. The potential advantage of targeting the GTP-bound ‘on’ state RAS is that this approach may be less susceptible to re-activation (or new mutations) from upstream pressure, such as from compensatory mutations or the overexpression of receptor tyrosine kinases leading to emerging resistance. Several companies have developed compounds which target the GTP-bound ‘on’ state of RAS, some of which have entered the clinic (NCT05379985). One such agent is RMC-6236, an oral tri-complex RAS^MULTI^ ‘on’ inhibitor that is now in phase I clinical trials. RMC-6236 non-covalently binds to an abundant intracellular chaperone protein, cyclophilin A (CypA), resulting in a binary complex that engages RAS ‘on’ to form a high-affinity, RAS-selective tri-complex that inhibits RAS binding to effectors [34]. As it does not rely on the cysteine binding site that the KRAS^G12C^ inhibitors do, this drug has been shown in preclinical models to have anti-tumor activity in multiple KRAS G12 isoforms including G12D, G12V and G12R, with the most common alterations identified in PDAC.

### 3.4. Adjuncts to RAS Inhibitors

In addition to the direct ‘on’/‘off’ RAS inhibitors, there are a variety of compounds in development targeting adjacent proteins which could act complementarily to RAS [4]. The main drug categories are compounds that target SOS1 or SHP2 and which are currently embarking on early-phase studies [35]. SOS1 is a major guanine nucleotide exchange factor (GEF) protein which vastly accelerates the GDP/GTP exchange rate on RAS, causing the activation of RAS [36]. SHP2 is a phosphatase with pleiotropic cellular functions, including the activation of RAS via mechanisms that are still not entirely clear [37]. The inactivation of SOS1 has been shown to decrease the survival of *RAS*-mutant tumor cells, but not of RAS wild-type cells [38]. The inhibition of SOS1 has been thought of as an attractive mechanism of RAS inhibition compared with direct RAS inhibitors, as most *RAS* mutants are still partially dependent on SOS1 for activation, and as this mechanism does not depend on targeting specific *KRAS* mutations. SOS1 inhibitors have made it to the clinic and are currently under investigation both alone and in combination strategies. BI-1701963 has been evaluated in a phase I setting in patients with *KRAS*-mutant advanced solid tumors, with 7 of 31 (23%) experiencing stable disease [39]. The combination therapy of a MEK inhibitor with BI-3406, another SOS1 inhibitor, blocks the negative feedback inhibition of SOS1 via MEK→ERK signaling, resulting in sustained pathway inhibition and potentiating the benefit of SOS1 inhibition. A phase I clinical trial of this SOS1 inhibitor in combination with trametinib, a MEK inhibitor, is ongoing (NCT04294160, NCT03989115). Experience with SHP2 inhibitors is more limited, although RMC-4630, an oral inhibitor of SHP2, has demonstrated preliminary efficacy in a KRAS^G12C^-mutant NSCLC population with a DCR of 71% (five of seven patients) [40].

The resulting shift from the active GTP to the inactive GDP-bound state following treatment with SOS1 and SHP2 inhibitors has led to enthusiasm for a combination therapy of these agents with mutant-specific KRAS inhibitors, such as KRAS^G12C^ inhibitors that rely on the GDP-bound state. Preclinical data have shown synergistic anti-cancer and improved anti-tumor efficacy for a combination of the SHP2 inhibitor and KRAS^G12C^ inhibitor and the combination of a SOS1 inhibitor with a KRAS^G12C^ inhibitor [41,42].

Other, more traditional, adjunctive targets are the upstream receptor kinases or the downstream effector network. As these compounds enter the clinic, it will be a major challenge to incorporate them in a rational way. Clinical trial design will be challenged by necessity, as it is unlikely that a ‘one size fits all’ approach will apply, even if enrolment is targeted narrowly.

### 3.5. Selective Downstream Inhibition

Before the recent advent and promise of direct RAS inhibitors, the dominant question was whether RAS inhibition could be achieved indirectly by blocking downstream nodes such as RAF or MEK. A whole series of clinical trials evaluated this strategy, but unfortunately it proved unsuccessful [43]. Early trials attempting to inhibit MEK1/2 in metastatic PDAC failed to demonstrate any clinical benefit alone or in combination with chemotherapy [10,44].

The explanations for this lack of activity are likely diverse—insufficient target inhibition and lack of a therapeutic window because of toxicity, in addition to the large number of possibilities for redundancy and feedback loops and crosstalk across multiple effectors. There is also the issue of tumor heterogeneity and the lack of attention paid to this in trial enrolment, in part due to lack of understanding of the specificity and mechanism of action of the downstream inhibitors. For example, clinically used RAF inhibitors enhance RAF kinase homo- and heterodimerization, leading to the paradoxical activation of ERK signaling [45]. The homo- and heterodimerization of the RAF kinases BRAF and CRAF significantly increase their catalytic activities. The binding of RAF molecules to active RAS drives RAF dimerization by inducing conformational changes bringing RAF molecules into proximity with each other [46]. Due to allosteric interactions between protomers in the RAF dimer, inhibitor binding to the first protomer in a dimer strongly decreases the affinity of the second protomer to the inhibitor. In this constellation, the drug-bound RAF protomer allosterically activates the drug-free protomer, causing paradoxical pathway activation and drug resistance [47]. As oncogenic RAS proteins are effective drivers of RAF kinase dimerization, *RAS* mutations lead to intrinsic or acquired resistance to RAF inhibitors. Overcoming dimerization-induced resistance to RAF inhibitors could—despite the negative experience of this indirect approach—lead to an effective anti-RAS therapy. Recently, a mathematical model that can simulate these resistance scenarios, allowing a thorough in silico analysis of the relevant mechanisms and ways to overcome them, has been developed. The results showed that resistance caused by RAF dimerization can be effectively broken by using two structurally different RAF inhibitors that synergize to block signaling by RAF dimers, as well as by oncogenic RAS [47]. A clinical trial (NCT05068752) that—based on this mathematical model—combines two different RAF inhibitors, vemurafenib and sorafenib, has recently started recruiting.

### 3.6. Autophagy

KRAS is also a central regulator of metabolism, including autophagy, a process of cellular recycling which protects PDAC cells from the cytotoxic effects of KRAS pathway inhibition and allows them to proliferate [48]. Interestingly, the inhibition of KRAS→RAF→MEK→ERK signaling elicits autophagy, and this process has been suggested as a mechanism of resistance to the downstream inhibition of MEK [48]. Therefore, inhibitors of autophagy have garnered great interest as a means to overcome this resistance mechanism. One study combining hydroxychloroquine with chemotherapy suggested an improved response rate, though no difference in survival benefit [49]. Combining downstream MEK inhibition with an inhibitor of autophagy resulted in pre-clinical and clinical responses, with case reports describing two cases of trametinib plus hydroxychloroquine used in the treatment of *KRAS*-mutated cancers achieving clinically meaningful benefit [50]. Various iterations of this approach are currently undergoing evaluation in the clinic.

### 3.7. RNA Interference (RNAi) Approaches

RNAi techniques have been under investigation as a tool to silence the expression of oncogenes and signaling effectors [51]. Various RNAi approaches are also being evaluated in PDAC. Earlier reports have demonstrated that the silencing of KRASG12V by retroviral short interfering RNA (siRNA) results in anti-cancer activity in a PDAC xenograft model [52]. Although there is significant interest, and preclinical evidence suggests a benefit, there has been no success in the clinic to date [53]. More recently, engineered vesicles have been developed as a delivery vehicle via a CD47-mediated uptake or biodegradable implants inserted into the tumor [54,55]. In an early-phase study, this was implanted directly into the tumor via endoscopic intervention in combination with systemic chemotherapy (NCT01188785). This demonstrated an encouraging PFS with no new safety signals [54]. This has led to phase II studies which are now evaluating a single dose of this siRNA in combination with chemotherapy (NCT01676259). Stromal-cell-derived engineered vesicles containing KRAS G12D siRNAs that present CD47 have also been developed and are now being tested in the clinic (NCT03608631) [56].

### 3.8. Immune Approaches

#### 3.8.1. Adoptive Cell Therapies

One proposed method for overcoming the classical barriers of reduced immune infiltration in KRAS-driven PDAC is the use of tumor-infiltrating lymphocytes (TILs) as T cell therapy. This concept of extracting TILs from a patient’s tumor, activating them and expanding them ex vivo before reinfusing them back into a lymphodepleted patient has been exploited in other solid KRASG12D-mutant tumors and is under investigation in PDAC [57,58].

The potential—and one of the problems—for adoptive cell therapies was graphically illustrated in an NEJM case report of a patient with mutant *KRAS^G12D^* metastatic colorectal cancer [57]. Polyclonal CD8+ tumor-infiltrating lymphocytes were obtained, and, following infusion, objective regression was observed. However, this case also highlighted the issue of emergent resistance, in that one of the metastatic lesions was found to have lost the chromosome 6 haplotype encoding the HLA-C*08:02 class I major histocompatibility complex (MHC) molecule that was targeted by the infused T cells. Loss of expression of this molecule provided a direct mechanism of tumor immune evasion. However, a number of different strategies, including T cell receptor therapy, chimeric antigen receptor (CAR-T) therapy and NK cell therapy, are being evaluated with the aim of improving this type of immunotherapy. CAR therapies have been developed as a tool to overcome MHC-based resistance and have been a success in hematological malignancies where both the presence of a ubiquitous target and the accessibility of tumor cells favored this therapeutic strategy [59]. A number of proposed neoantigens in *KRAS*-mutated cancers have been identified, including CEA, MUC1 mesothelin and CD24 [60]. Unfortunately, a phase I trial of CAR-T cells expressing chimeric anti-MSLN and CD3-ζ with 4-1BB costimulatory domains produced limited clinical activity [61]. Similarly, promising preclinical data targeting CEA with CAR-T cells failed to demonstrate the hoped-for clinical benefits [62].

#### 3.8.2. Vaccines

The recent COVID pandemic accelerated the development of mRNA vaccines as a potential means of inducing specific targeted immunity, ultimately negating the need to directly inhibit KRAS via other mechanisms. The implications for cancer vaccine development are also clear, and several compounds are in early-phase testing. Despite significant efforts, very few therapeutic cancer vaccines have made it to clinical practice [63]. Other novel vaccines are currently in development, with a number of different vaccine types available, including peptide-based, dendritic-cell-based or mRNA vaccines. Peptide-based vaccines attempt to capitalize on neoantigens to elicit a strong tumor-specific immune response. A number of strategies have been developed using a personalized approach to identify specific neoantigens on patients’ biopsies, in order to create a peptide-based vaccine. Synthetic long peptides with KRAS G12D have been tested, resulting in an increase in CD4+ and CD8+ T cells, with accompanying tumor responses evident [64]. Other efforts have focused on modifying the peptide structure to improve delivery. Amphiphilic vaccines exploit the fact that larger proteins such as albumin almost exclusively transit from subcutaneous tissue into lymph, unlike smaller peptides, which enter the bloodstream and are rapidly distributed and cleared. Combination with a CpG oligonucleotide adjuvant aids uptake specifically to the lymph nodes, leading to a 30- to 50-fold increase in T cell and antibody responses to peptide vaccines [65,66]. ELI-002 is an amphiphilic vaccine composed of lipophilic modified RAS peptides that is currently being evaluated in a multicenter trial in patients with *RAS*-mutated cancers [67].

Dendritic cells and other modified antigen-presenting cells have demonstrated increased immune responses and anti-tumor activity in PDAC models and are under investigation in clinical trials including patients with PDAC, following initial reports of clinical benefit in patients with NSCLC [68] (NCT03329248).

mRNA vaccines can express multiple antigens at once, theoretically allowing for a stronger and more sustained specific immune response. Many of the studies of mRNA vaccine therapy are now being tested in combination with immune checkpoint inhibitors such as the anti-PD-1 therapy pembrolizumab (NCT03897881). The underlying idea is that the addition of immune checkpoint inhibitors will remove any barriers to immune activation and identification. mRNA-5671 contains KRAS G12D-, G12V-, G13D- and G12C-specific peptides and is currently under investigation in combination with pembrolizumab in phase I clinical trials in patients with *KRAS*-mutant advanced cancer, including those with PDAC (NCT03948763).

#### 3.8.3. Immune Checkpoint Inhibitors

Oncogenic KRAS alterations result in immune evasion, as described above [6]. Although immune checkpoint inhibitors blocking PD-1, PD-L1 or CTLA-4 have demonstrated remarkable benefits in certain cancers, they have failed to show any improvement in outcomes for patients with PDAC, with a notable exception of those patients with mismatch repair protein deficiency [69,70,71]. As a result of this underwhelming activity, the focus has shifted to identify combination strategies in order to unlock the benefits of immune checkpoint blockade. PDAC features a high expression level of co-stimulatory inhibitory molecules that predict a reduced response to immunotherapy and worse clinical outcomes [72]. Much research has focused on identifying and targeting these co-stimulatory molecules, such as CD47, LAG-3 or TIM-3, to target alongside PD-1, including in patients with KRAS alterations. The direct inhibition of KRAS with sotorasib in combination with anti-PD-1 therapy has shown improved responses in mouse models, suggesting that the immune microenvironment can be augmented through the inhibition of KRAS [73].

## 4. Conclusions

In a relatively short space of time, there has been a major change in attitudes to targeting RAS in cancer in general and—given the dominant role this oncoprotein plays—in PDAC in particular. While there has been much recent success with direct KRAS inhibitors in other malignancies, the contrasting KRAS mutation variants in PDA have been largely resistant to direct targeting. Efforts, therefore, are being made not just to target the RAS protein directly, but also to target upstream regulator and downstream effector molecules, as well as the tumor microenvironment and immune system that result from deregulated RAS signaling. The application of mathematical modeling to resistance mechanisms has led to novel combination strategies that will hopefully improve on the durability of response to targeted therapy. While innovative vaccine treatments coupled with immune checkpoint blockade may offer some hope at implementing immunotherapy in a disease traditionally immune-cold, the intra-tumor heterogeneity still represents a significant barrier to overcome. Sophisticated approaches utilizing RNAi to directly deliver targeted therapies have been shown to be safe, and capable of inhibiting KRAS with few to no off-target side effects [54]. Other advanced techniques, however, such as adoptive cell therapies, have yet to demonstrate any compelling benefit. With a host of new agents entering clinical investigation, future clinical efforts will now focus on either the combination or the sequence of these treatments. The KRAS^G12C^ story and the development of targeted therapy for patients harboring those specific alterations demonstrates that, with continued effort, other therapeutic strategies will become fruitful.

## Data Availability

Not applicable.
